# Radiomics for differentiation of gliomas from primary central nervous system lymphomas: a systematic review and meta-analysis

**DOI:** 10.3389/fonc.2024.1291861

**Published:** 2024-02-14

**Authors:** Alexandru Garaba, Nummra Aslam, Francesco Ponzio, Pier Paolo Panciani, Waleed Brinjikji, Marco Fontanella, Lucio De Maria

**Affiliations:** ^1^ Department of Surgical Specialties, Radiological Sciences, and Public Health, University of Brescia, Brescia, Italy; ^2^ Interuniversity Department of Regional and Urban Studies and Planning, Politecnico di Torino, Torino, Italy; ^3^ Department of Neurosurgery and Interventional Neuroradiology, Mayo Clinic, Rochester, MN, United States; ^4^ Department of Clinical Neuroscience, Geneva University Hospitals (HUG), Geneva, Switzerland

**Keywords:** radiomics, gliomas, primary central nervous system lymphomas, systematic review, meta-analysis

## Abstract

**Background and objective:**

Numerous radiomics-based models have been proposed to discriminate between central nervous system (CNS) gliomas and primary central nervous system lymphomas (PCNSLs). Given the heterogeneity of the existing models, we aimed to define their overall performance and identify the most critical variables to pilot future algorithms.

**Methods:**

A systematic review of the literature and a meta-analysis were conducted, encompassing 12 studies and a total of 1779 patients, focusing on radiomics to differentiate gliomas from PCNSLs. A comprehensive literature search was performed through PubMed, Ovid MEDLINE, Ovid EMBASE, Web of Science, and Scopus databases. Overall sensitivity (SEN) and specificity (SPE) were estimated. Event rates were pooled using a random-effects meta-analysis, and the heterogeneity was assessed using the χ2 test.

**Results:**

The overall SEN and SPE for differentiation between CNS gliomas and PCNSLs were 88% (95% CI = 0.83 – 0.91) and 87% (95% CI = 0.83 – 0.91), respectively. The best-performing features were the ones extracted from the Gray Level Run Length Matrix (GLRLM; ACC 97%), followed by those obtained from the Neighboring Gray Tone Difference Matrix (NGTDM; ACC 93%), and shape-based features (ACC 91%). The 18F-FDG-PET/CT was the best-performing imaging modality (ACC 97%), followed by the MRI CE-T1W (ACC 87% - 95%). Most studies applied a cross-validation analysis (92%).

**Conclusion:**

The current SEN and SPE of radiomics to discriminate CNS gliomas from PCNSLs are high, making radiomics a helpful method to differentiate these tumor types. The best-performing features are the GLRLM, NGTDM, and shape-based features. The 18F-FDG-PET/CT imaging modality is the best-performing, while the MRI CE-T1W is the most used.

## 1 Introduction

Radiomics is a rapidly expanding field that extracts quantitative information from medical images that is then analyzed using artificial intelligence (AI) techniques such as machine learning (ML) and its subcategory, deep learning (DL) to define radiomics features, which may include tissue heterogeneity, texture, shape, and gray intensity (1, 2). These features can help distinguish between tumor types, thus aiding in proper diagnosis and treatment planning.

Distinguishing between central nervous system (CNS) gliomas and primary central nervous system lymphomas (PCNSLs) can be difficult due to their similar radiological appearance. Both tumors can exhibit imaging features such as heterogeneity, necrosis, and contrast enhancement (3). Current clinical standards involve a multifaceted approach, combining imaging studies, such MRI, and histopathological analysis through biopsy. Despite advancements in conventional diagnostic methods, there are several challenges. Biopsy procedures, while informative, may not always be feasible or carry inherent risks. Additionally, the reliance on imaging studies may lead to overlapping features, complicating the interpretation and contributing to misdiagnoses. Accurate diagnosis is essential for patient care, as it drives different treatment approaches (4, 5). Radiomics has shown promising results in distinguishing between them, potentially improving patient outcomes (6).

Despite the promising results, a meta-analysis of the current literature is required to determine the effectiveness of radiomics in distinguishing gliomas from PCNSLs. We conducted a systematic review and meta-analysis of studies that used radiomics to differentiate between these tumors. We aimed to evaluate the overall diagnostic performance of radiomics in differentiating these two tumor types and identify the most effective radiomics variables to pilot future models.

The findings of this study may be employed in clinical practice and can potentially improve patient outcomes allowing accurate diagnosis and treatment planning.

## 2 Materials and methods

### 2.1 Literature search

The systematic review was performed according to the Preferred Reporting Items for Systematic Reviews and Meta-Analysis (PRISMA) guidelines (7). A comprehensive literature search of the databases PubMed, Ovid MEDLINE, Ovid EMBASE, Web of Science, and Scopus was designed and conducted by an experienced librarian with input from the authors. These databases were selected for their extensive coverage of relevant medical literature, ensuring a thorough retrieval of studies related to radiomics in the context of CNS gliomas and PCNSLs. The following research string was used: “radiomics AND glioma AND lymphoma”. The studies were found using the Medical Subject Heading (MeSH) terms and Boolean operators. A search filter was set to show only publications over the designated period. The search was limited to articles published between 2012 and 2022. The first literature search was performed on June 2, 2023, and the search was updated on July 20, 2023.

Two Authors (L.D.M. and F.P.) determined the inclusion criteria for the studies in the literature search process. The following inclusion criteria were used: 1) case series including at least 10 patients, 2) studies reporting on radiomics for the differential diagnosis of CNS gliomas and PCNSLs, 3) availability of performance data for differentiation of these tumors, 4) studies reporting exclusively histologically proven CNS gliomas and PCNSLs, and 5) inclusion of both multi-center and single-center studies. Exclusion criteria were: 1) case reports or review studies, 2) studies reporting on AI-based models other than radiomics, 3) studies on radiomics differentiation of other tumor types, and 4) studies not reporting performance data of the radiomics model.

The list of identified studies was imported into Endnote X9, and duplicates were removed. The search results were checked by two independent researchers (F.P. and W.B.) with experience according to the inclusion and exclusion criteria. A third blinded reviewer (L.D.M.) resolved all disagreements. Then, eligible articles were subject to full-text screening. Reference lists of selected studies were also reviewed to identify additional relevant studies.

### 2.2 Data extraction

For each study, we abstracted the following baseline information: year of publication, total number of patients, distribution of patients per tumor type, and magnetic resonance imaging (MRI) protocol. As for the radiomics models, we collected information about the AI sub-category [i.e. ML or DL], classification algorithms [i.e. logistic regression (LR), support vector machine (SVM), naïve bayes (NB), k-nearest neighbor (kNN), multilayer perceptron (MLP), random forest (RF), adaptive boosting (AdaBoost), elastic net regression (ENR), linear discriminant analysis (LDA), convolutional neural network (CNN), least absolute shrinkage and selection operator regression (LASSO regression), others], best-performing classifiers, best-performing features [i.e. textural, geometrical or morphological, voxel intensities-based, others], best-performing MRI sequences [i.e. T1-weighted (T1W), contrast-enhanced T1 weighted image (CE-T1W), T2-weighted (T2W), T2 weighted fluid-attenuated inversion recovery (T2-FLAIR), diffusion-weighted imaging (DWI), dynamic susceptibility contrast (DSC), perfusion-weighted imaging (PWI), T1-weighted fast field echo (T1-FFE), fluorine-18 fluorodeoxyglucose positron emission tomography/computed tomography (18F-FDG-PET/CT), computed tomography (CT) scans], and application of cross-validation analysis [i.e. yes or no]. Regarding the performance of the models, we extracted data on the resultant sensitivity (SEN) and specificity (SPE), accuracy (ACC), positive predictive value (PPV), and negative predictive value (NPV).

In studies with overlapping patient populations written by the same Authors or Institution, we only included the largest or most complete dataset. In cases where outcomes were separated by study cohorts, we abstracted performance outcomes of validation or test cohorts to perform our meta-analysis.

### 2.3 Outcomes

Our primary outcomes were the overall SEN, SPE, and summary receiver operating characteristics (SROC) curve of radiomics for the differentiation of CNS gliomas from PCNSLs. Bivariate analyses by discrimination task between CNS gliomas and PCNSLs were conducted. In terms of the performance of the models, we also looked at the ACC, PPV, and NPV.

As secondary outcomes, we performed a moderator analysis to provide a thorough explanation of the possible sources of heterogeneity in observed effect sizes. In this regard, we analyzed the impact of the following variables on the performance of the proposed radiomics models: year of development, cohort size, AI sub-category, best-performing classifiers, features, MRI sequences, the presence or absence of external test sets in the studies, and application of cross-validation. These variables were also studied quantitatively to picture the current trends of radiomics models for differentiating these tumor types.

### 2.4 Study risk of bias assessment

We modified the Newcastle-Ottawa Scale (NOS) to assess the methodologic quality of the studies included in our meta-analysis (8). This tool, originally designed for use in comparative studies with a control group, was adapted to our study. As there was no control group in our studies, we assessed their methodologic quality based on selected items from the scale, focusing on the following questions: 1) Was the study retrospective or prospective? 2) Were there clearly defined inclusion and exclusion criteria? 3) Did the study include all patients or consecutive patients vs. a selected sample? 4) Were outcomes reported? 5) Was clinical follow-up satisfactory, thus allowing ascertainment of all outcomes? (9).

### 2.5 Statistical analysis

For the purpose of the meta-analysis, we considered the total number of patients included in each study’s test or validation dataset. Data from primary studies were reported in a 2×2 contingency table consisting of true positive (TP), false positive (FP), false negative (FN), and true negative (TN) based on the concordance between biopsy results and the radiomics tool predictions. Such a table served as input for the R-package *mada* (10), used for modeling the joint estimates of SEN and SPE and their 95% confidence intervals (CIs). Event rates were pooled across studies using a random-effects meta-analysis, and the χ^2^ test was performed to assess the heterogeneity of SEN and SPE, considering the null hypothesis as equality in each case.

SEN and SPE depend on each other via a cut-off value: as the cut-off is varied to, say, increase the sensitivity, the specificity often decreases. Hence, the two quantities are negatively correlated. Thus, we opted for the bivariate meta-analytic strategy via *mada* routines, which leverage a linear mixed model derived from the approach described by Ardens et al. (11).

To better show the diagnostic performance of AI-based radiomics tools, we made the following further figures of merit: 1) univariate graphics in the form of forest plots for both SEN and SPE; 2) endpoints of interest with individual confidence regions; 3) SROC curve seeking to combine ROC curves of primary studies. In these last two graphical outcomes, the coordinates of the endpoints of interest are in the form of [SEN] and [1 – SPE], the latter better known as the false positive rate (FPR).

## 3 Results

### 3.1 Literature review

**Figure 1:**
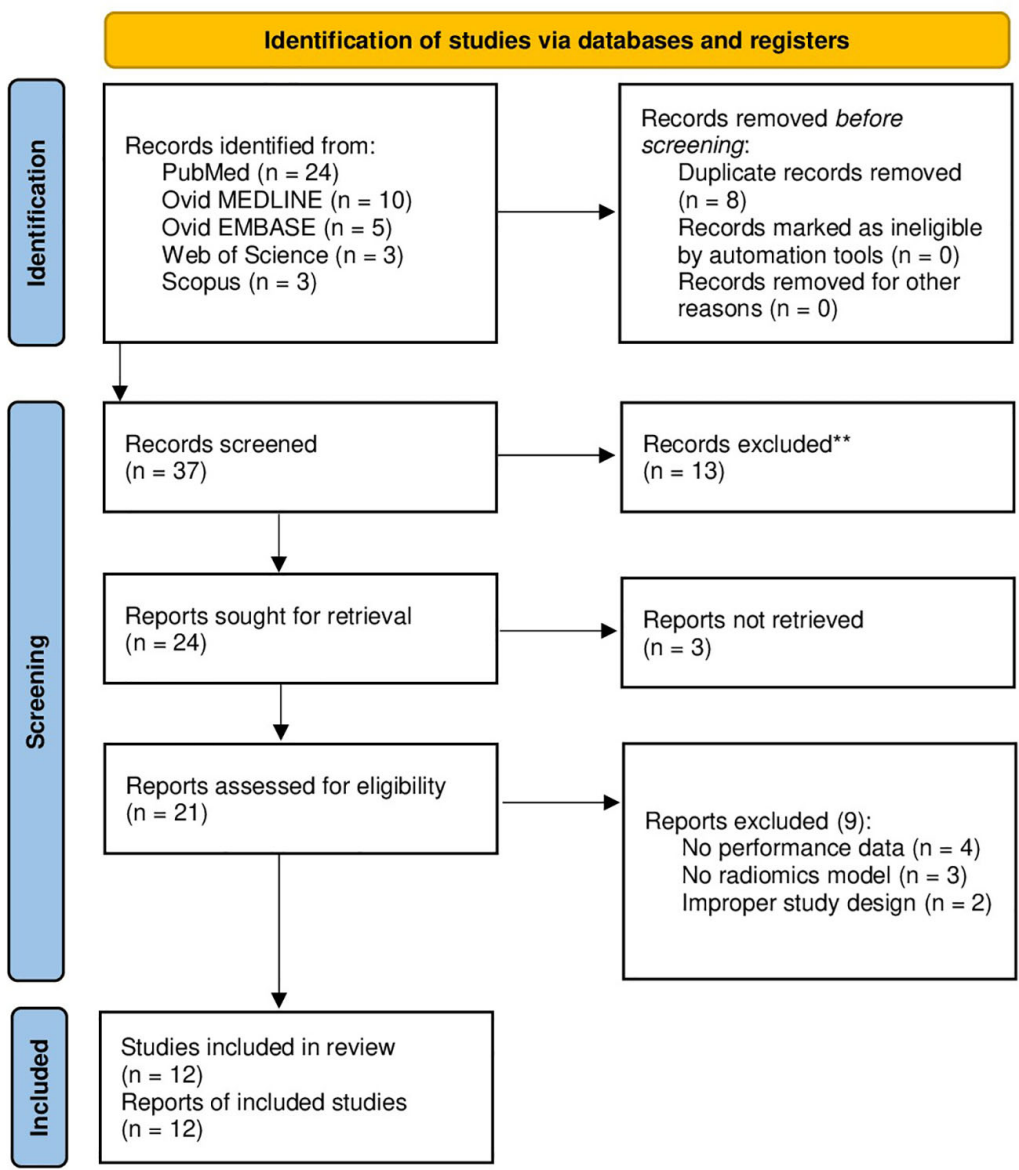
PRISMA flow diagram depicting the literature search process. PRISMA, preferred reporting items for systematic reviews and meta-analysis.

A total of 37 papers were identified after duplicate removal. After title and abstract analysis, 21 articles were identified for full-text analysis. Eligibility was ascertained for 12 articles (12–23) The remaining 9 articles were excluded for the following reasons: 1) studies not reporting data on radiomics performance for differentiation of gliomas from PCNSLs (4 articles), 2) studies reporting on AI-based models other than radiomics (3 articles), 3) improper study design (2 articles). All studies included in the analysis had at least one or more outcome measures available for one or more of the patient groups analyzed. **Figure 1** shows the flow chart according to the PRISMA statement (7).

### 3.2 Baseline and radiomics data

A total of 1779 patients were included in the meta-analysis. Most studies were published in 2018 (4), followed by 2021 (3), 2022 (2), 2019 (2), and 2020 (1). The smallest study included 77 patients (16), while the largest included 289 patients (22). Differentiation between CNS gliomas and PCNSLs was reported for 1779 patients, of which 1063 had gliomas (59,75%), and 716 had PCNSLs (40,25%). A number of 10 studies included different MRI sequences and the most common was the CE-T1W (24,24%), followed by DWI (21,21%), T2-FLAIR (18,18%), and T2W (15,15%). Other MRI modalities were T1W (12,12%), PWI (3,03%), DSC (3,03%), and T1-FFE (3,03%). In addition to the MRI studies, there was also one study conducted using 18F-FDG-PET/CT, and one study using CT.

A number of 8 studies reported on ML (66,67%), three articles reported on DL (25%), and there was 1 hybrid study (8,33%).

When it comes to studies utilizing DL, one employed a well-defined CNN model, specifically DenseNet-121 (22), while the remaining studies utilized custom-designed CNN architectures that were described within their respective articles (13, 20). Notably, one of these studies did not specify the architecture used (23).

As for the classifiers, SVM was the most adopted (13,20%), followed by RF (9,43%), LR (9,43%), AdaBoost (7,54%), and others. A summary of the included studies is provided in **Table 1**.

**Table 1:** Summary of studies.

Author, Journal, Year	Dataset (No.)	Gliomas (No.)	PCNSLs (No.)	Imaging protocol	Method	Classifiers
Bathla, European Radiology, 2021 (12)	94	60	34	T1W, CE-T1W, T2W, T2-FLAIR, DWI	ML	Linear Regression, LR, RR, ENR, LASSO, NN, SVM with a polynomial kernel, SVM with a radial kernel, MLP, RF, GBRM, AdaBoost
Chen, The International Journal of Neuroscience, 2018 (13)	96	66	30	CE-T1W	DL	SVM
Kang, Neuro-Oncology 2018 (14)	196	119	77	T1W, CE-T1W, T2W, T2-FLAIR, DWI, PWI	ML	K-NN, NB, DT, LDA, RF, AdaBoost, Linear SVM, RBF kernel SVM
Kim, Neuroradiology, 2018 (15)	143	78	65	T1-FFE, T2W, DWI, T2-FLAIR	ML	LR, SVM, RF
Kong, Neuroimage Clinical, 2019 (16)	77	53	24	18F-FDG-PET/CT	ML	DT
Lu, Frontiers in neurology, 2022 (17)	101	51	50	CT scans	ML	LR, RF, DT, K-NN, SVM, NB
Lv, Journal of Neurosurgery, 2022 (18)	103	68	35	CE-T1W	ML	k-NN, GNB, RF, LR, SVM, MLP, AdaBoost
Priya, Neuroradiol J., 2021 (19)	143	97	46	T1W, CE-T1W, T2W, T2-FLAIR, DWI, PWI	ML	Linear regression, multinomial logistic, RR, elastic net, LASSO, NN, SVM with a polynomial kernel, SVM with a radial kernel, MLP, RF, GBRM, AdaBoost
Wu, IEEE Transanctions On Medical Imaging, 2018 (20)	102	70	32	CE-T1W, T2W	ML, DL	Sparse Representation, CNN
Xia, Journal of Magnetic Resonance Imaging, 2020 (21)	240	129	111	CE-T1W, T2-FLAIR, DWI	ML	LASSO, Multi-variable LR
Xia, Journal of Magnetic Resonance Imaging, 2021 (22)	289	153	136	T1W, T2-FLAIR, DWI	DL	CNN
Yun, scientific reports, 2019 (23)	195	195	119	CE-T1W, DWI	DL	MLP

PCNSL, primary central nervous system lymphoma; T1W, T1-weighted; CE-T1W, contrast-enhanced T1 weighted image; T2W, T2-weighted; T2-FLAIR, T2 weighted fluid-attenuated inversion recovery; DWI, diffusion-weighted imaging; PWI, perfusion-weighted imaging; T1-FFE, T1-weighted fast field echo; CT, computed tomography; 18F-FDG-PET/CT, fluorine-18 fluorodeoxyglucose positron emission tomography/computed tomography; ML, machine learning; DL, deep learning; LR, logistic regression; RR, ridge regression; ENR, elastic net regression; LASSO, least absolute shrinkage and selection operator; NN, neural network; SVM, support vector machine; MLP, multilayer perceptron; RF, random forest; GBRM, generalized boosted regression model; AdaBoost, adaptive boosting; k-NN, k-nearest neighbor; NB, naïve bayes; DT, decision tree; LDA, linear discriminant analysis; RBF, radial basis function; GNB, gaussian naïve bayes; CNN, convolutional neural network.

### 3.3 Primary outcomes

**Figure 2:**
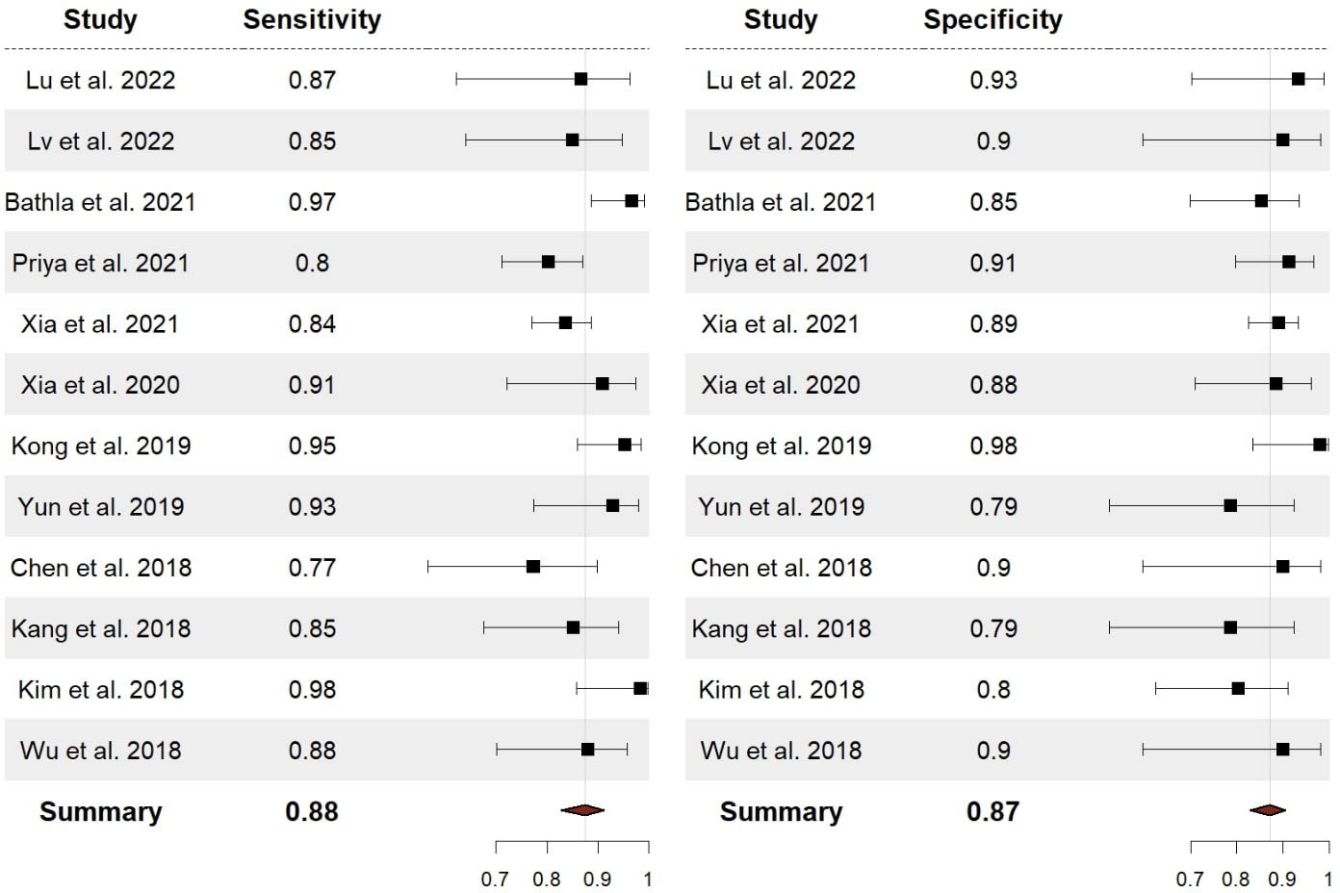
Forest plots with overall SEN and SPE for discrimination between CNS gliomas and PCNSLs. SEN, sensitivity; SPE, specificity; CNS, central nervous system; PCNSL, primary central nervous system lymphoma.

**Figure 3:**
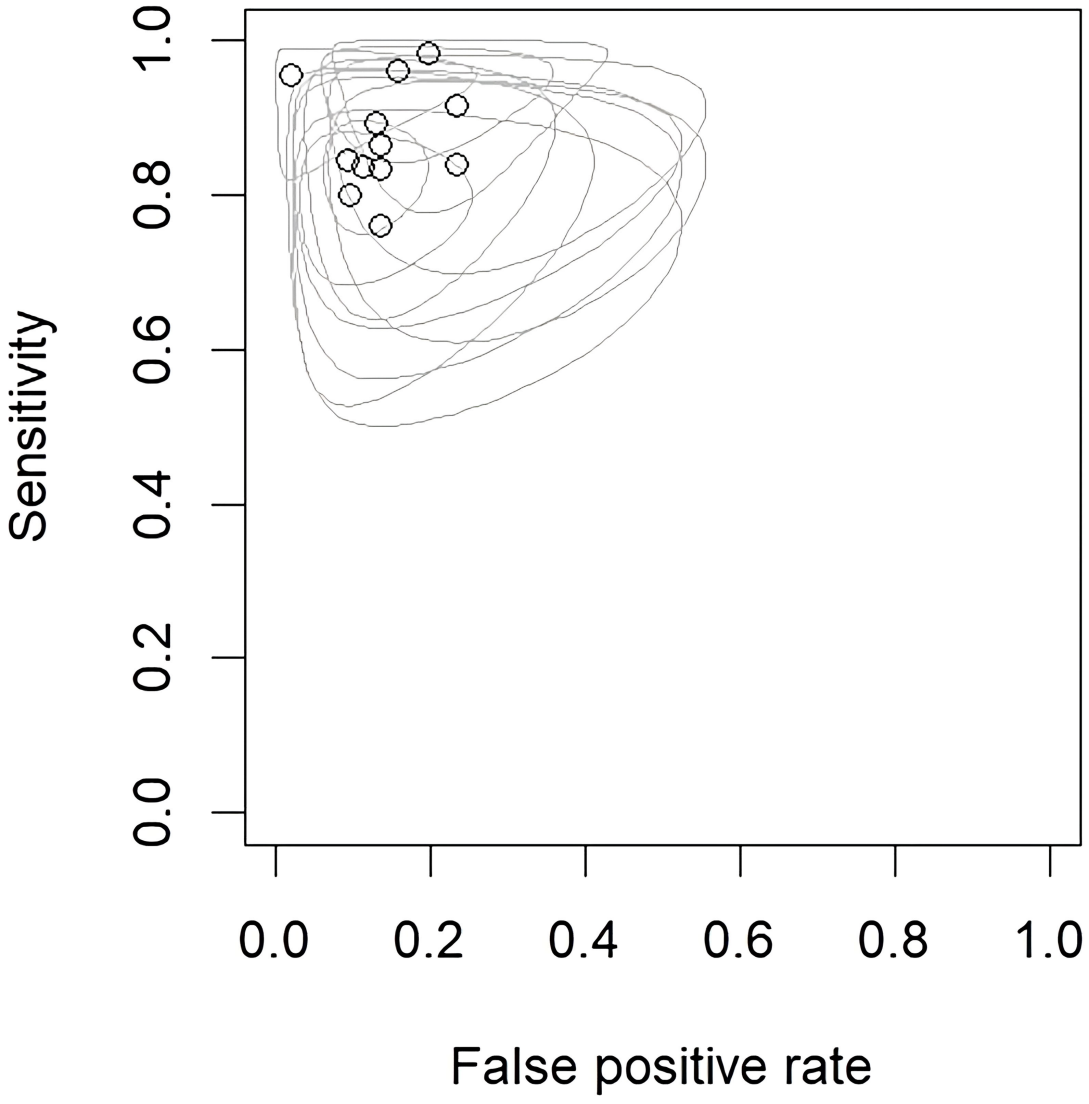
Endpoints of interest with individual confidence regions for differentiation between CNS gliomas and PCNSLs. CNS, central nervous system; PCNSL, primary central nervous system lymphoma.

**Figure 4:**
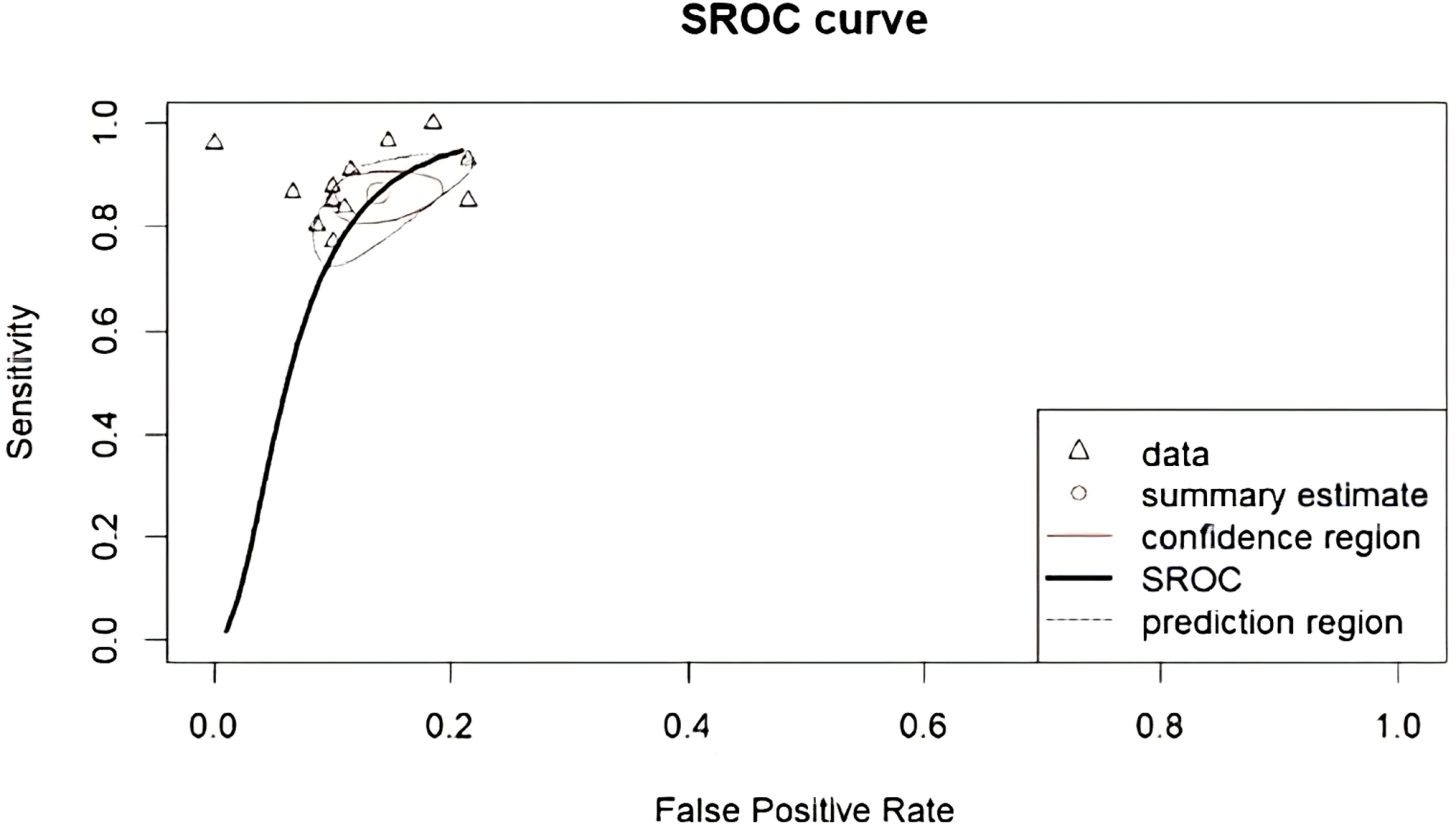
Corresponding SROC curve for differentiation between CNS gliomas and PCNSLs. SROC, summary receiver operating characteristic; CNS, central nervous system; PCNSL, primary central nervous system lymphoma.

The performance of radiomics to discriminate between CNS gliomas and PCNSLs was reported for a total of 993 patients composing the validation or test datasets of the studies included in our meta-analysis. The Overall SEN and SPE were 88% (95% CI = 0.83 – 0.91) and 87% (95% CI = 0.83 – 0.91). **Figure 2** shows the SEN and SPE forest plots of the bivariate analyses for discrimination between CNS gliomas and PCNSLs. **Figure 3** provides the individual confidence regions and **Figure 4** the corresponding SROC curve for the differentiation task. Specifically, the summary estimate coordinates of the SROC curve for CNS gliomas vs. PCNSLs were [0.87; 0.14]. The ACC of the included studies ranged from 83% (14) to 97% (16), the PPV from 85% (15) to 100% (16), and the NPV from 74% (14) to 100% (15). **Table 2** summarizes the performance data of the radiomics models analyzed.

**Table 2:** Summary of performance data of the radiomics models.

Author, Journal, Year	SEN	SPE	ACC	PPV	NPV
Bathla, European Radiology, 2021 (12)	0,97	0,85	0,93	0,93	0,94
Chen, The International Journal of Neuroscience, 2018 (13)	0,77	0,90	0,91	0,98	0,68
Kang, Neuro-Oncology 2018 (14)	0,85	0,79	0,83	0,91	0,74
Kim, Neuroradiology, 2018 (15)	0,98	0,80	0,91	0,85	1,00
Kong, Neuroimage Clinical, 2019 (16)	0,95	0,98	0,97	1,00	0,92
Lu, Frontiers in neurology, 2022 (17)	0,87	0,93	0,90	0,94	0,87
Lv, Journal of Neurosurgery, 2022 (18)	0,85	0,90	0,90	0,95	0,79
Priya, Neuroradiol J., 2021 (19)	0,80	0,91	0,88	0,96	0,69
Wu, IEE Transanctions On Medical Imaging, 2018 (20)	0,88	0,90	0,95	0,98	0,79
Xia, Journal of Magnetic Resonance Imaging, 2020 (21)	0,91	0,88	0,92	0,90	0,93
Xia, Journal of Magnetic Resonance Imaging, 2021 (22)	0,84	0,89	0,87	0,90	0,83

SEN, sensitivity; SPE, specificity; ACC, accuracy; PPV, positive predictive value; NPV, negative predictive value.

### 3.4 Secondary outcomes

Our subgroup analysis could not evidence variables that significantly impact the performance of the models. Nonetheless, the best-performing features were those extracted from the Gray Level Run Length Matrix (GLRLM; ACC 97%) (16), followed by the ones provided by the Neighboring Gray Tone Difference Matrix (NGTDM; ACC 93%) (12) and then shape-based features (ACC 91%). As for the imaging modalities, the best-performing was the 18F-FDG-PET/CT (ACC 97%) (16), followed by the MRI CE-T1W reported in another 7 studies (ACC 87% (22) - 95% (20)). It’s worth mentioning that half of the included studies incorporated external test sets for evaluation (13–15, 18, 21, 23), while the remaining half solely relied on their internal datasets (12, 16, 17, 19, 20, 22). Additionally, most studies applied a cross-validation analysis (92%). **Table 3** summarizes the secondary outcomes.

**Table 3:** Cross-validation analysis, best-performing features, and best-performing imaging modalities per each study.

Author, Journal, Year	Cross-validation analysis	Best- performing features	Best- performing imaging modalities
Bathla, European Radiology, 2021 (12)	Yes	NGTDM	CE-T1W
Chen, The International Journal of Neuroscience, 2018 (13)	Yes	NA	CE-T1W
Kang, Neuro-Oncology 2018 (14)	Yes	NA	DWI
Kim, Neuroradiology, 2018 (15)	Yes	Shape-based	NA
Kong, Neuroimage Clinical, 2019 (16)	Yes	GLRLM	18F-FDG-PET
Lu, Frontiers in neurology, 2022 (17)	Yes	NA	CT scans
Lv, Journal of Neurosurgery, 2022 (18)	No	NA	CE-T1W
Priya, Neuroradiol J., 2021 (19)	Yes	First order	CE-T1W
Wu, IEE Transanctions On Medical Imaging, 2018 (20)	Yes	NA	CE-T1W
Xia, Journal of Magnetic Resonance Imaging, 2020 (21)	Yes	NA	CE-T1W
Xia, Journal of Magnetic Resonance Imaging, 2021 (22)	Yes	NA	CE-T1W

NGTDM, neighboring gray tone difference matrix; GLRLM, gray level run length matrix; CE-T1W, contrast-enhanced T1-weighted image; DWI, diffusion-weighted imaging; 18F-FDG-PET/CT, fluorine-18 fluorodeoxyglucose positron emission tomography/computed tomography; CT, computed tomography; NA, Not Available.

**Figure 5:**
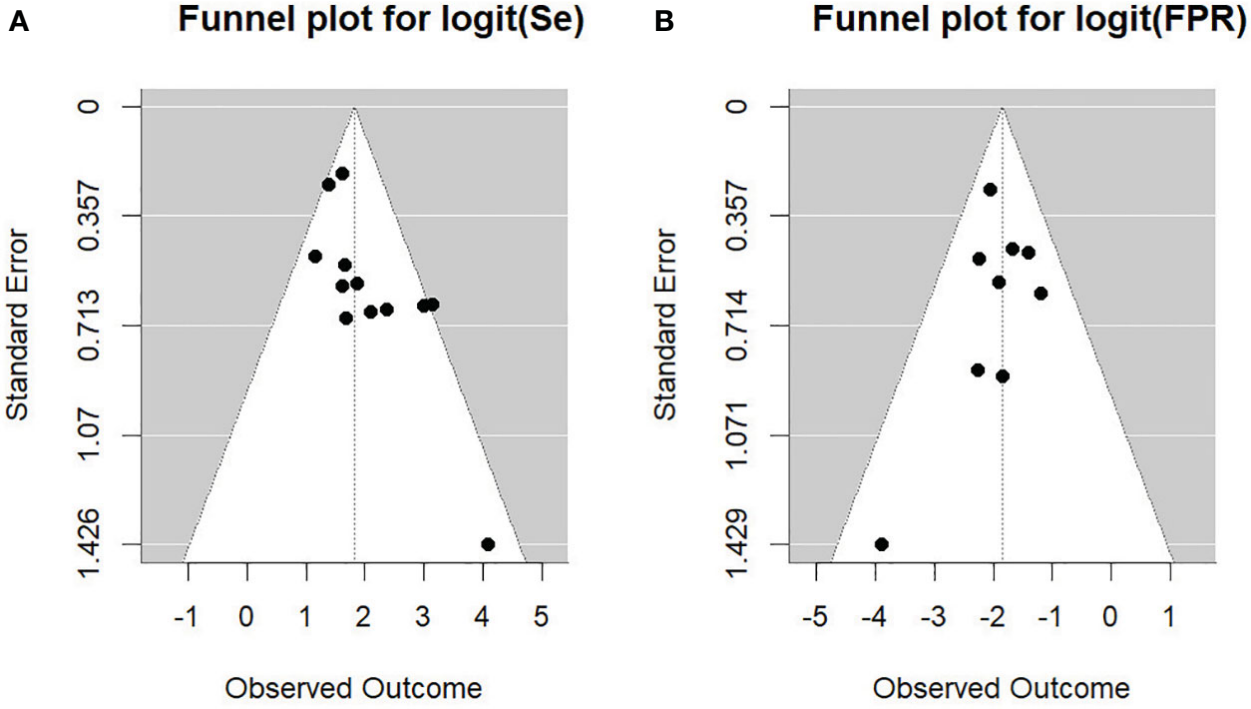
Funnel plots for the bivariate outcomes. **(A)** Se, sensitivity; **(B)** FPR, false positive rate.

We furthermore investigated the presence of potential publication bias, as well as small study effect, conducting the generalized Egger’s test, which can incorporate the correlation information intrinsic in the diagnostic test accuracy meta-analysis (24). The outcome of the test was not significant (p=0.052), indicating no asymmetry into the funnel plot. This aspect can be also gathered from **Figure 5** providing the Funnel plots for the bivariate outcomes of our diagnostic meta-analysis, namely the logit of the SEN and of the FPR (see **Figure 5** left and right respectively).

### 3.5 Study heterogeneity

The χ^2^ test suggested substantial heterogeneity of SEN (χ^2 = ^20.5, df=11, *P*=0.04) and SPE (χ^2 = ^21.3, df=11, *P*=0.03) for the CNS gliomas vs. PNCSLs differentiation task.

## 4 Discussion

In this study, we conducted a comprehensive meta-analysis to assess the performance of radiomics in discriminating between CNS gliomas and PCNSLs. A total of 12 eligible articles were included in the analysis, encompassing a dataset of 1779 patients. Most studies employed ML techniques, with SVM being the most used classifier.

Overall, radiomics demonstrated promising performance in discriminating between these two tumor types, with certain texture matrices, such as GLRLM and NGTDM, providing valuable information for the extraction of diverse radiomic features and showing higher accuracy in this discrimination process. Additionally, the utilization of 18F-FDG-PET/CT and CE-T1W MRI modalities yielded the most promising results.

### 4.1 Radiomics models

The studies included in the meta-analysis used two different AI techniques in the context of radiomics, namely ML and its subcategory, DL. These advanced computational techniques allowed researchers to extract high-dimensional imaging features from different modalities, including MRI and PET/CT scans, enabling a more comprehensive characterization of these tumors.

Among the AI models employed, ML was the most used approach, reflecting its well-established presence in medical imaging research. SVM emerged as the most adopted classifier, equipped with radial basis function or polynomial kernel in most of the cases. RF and LR were also frequently employed.

Furthermore, a noteworthy percentage of studies (25%) incorporated DL techniques, which indicates the growing interest and exploration of neural networks for radiomics analysis. DL models have demonstrated their potential to automatically learn and identify intricate patterns in medical images, potentially leading to more accurate and efficient tumor classification (25).

Interestingly, a hybrid study combined both ML and DL techniques, leveraging the complementary strengths of these approaches (20). Such integration of diverse AI methodologies may offer synergistic advantages in capturing complex imaging patterns and enhancing the overall predictive power of radiomics models (26).

The imaging modalities used in the included studies were primarily MRI-based, with the CE-T1W being the most common sequence. This preference can be attributed to the valuable contrast enhancement information provided by CE-T1W, which aids in identifying regions of abnormal vascularity and enhancing tumor boundaries. The DWI, T2-FLAIR, and T2W sequences were also frequently employed, each contributing unique information about the tumor’s cellular density, edema, and structural characteristics.

Additionally, two studies utilized alternative imaging techniques, namely 18F-FDG-PET/CT (16) and CT (17). These studies represent a broader exploration of radiomics beyond traditional MRI-based approaches, and they may offer unique insights into tumor metabolism and density, respectively.

The radiomics models developed in these studies showed promising results in differentiating CNS gliomas from PCNSLs and have the potential to contribute significantly to improved patient outcomes, aiding in accurate and timely diagnoses, as well as personalized treatment strategies.

### 4.2 Radiomics performance

In this meta-analysis, we evaluated the performance of radiomics in discriminating between CNS gliomas and PCNSLs using data from a total of 993 patients from various validation or test datasets included in the selected studies. The bivariate analyses yielded an overall SEN and SPE of 88% and 87%, respectively. The SROC curve further illustrated the differentiation task with summary estimate coordinates of [0.87; 0.14].

When comparing our radiomics-based results to other non-radiomic studies mentioned in the literature, we observed competitive performance. While it is essential to note that the studies included in our meta-analysis varied in terms of population size, methodology, and the specific radiomics features used, our overall SEN and SPE surpassed the reported diagnostic accuracy of some non-radiomic approaches.

For instance, a study used DWI in association with ADC to differentiate between CNS gliomas and PCNSLs, but the findings indicated a slightly lower diagnostic performance, with a SEN of 82% (95% CI 0.75-0.88) and a SPE of 87% (95% CI 0.84-0.90) (27). Similarly, in another study, the diagnostic efficacy of DWI was investigated for the same discrimination task, but it also exhibited lower performance, with SEN at 82% (95% CI 0.70-0.90) and SPE at 84% (95% CI 0.75-0.90) (28). DWI provides valuable insights into tissue microstructure by measuring the random motion of water molecules, offering a direct examination of cellular density and tissue organization. This information can be particularly useful for distinguishing between different tissue types and detecting subtle alterations in cellularity. However, DWI has its limitations, primarily related to its sensitivity to motion artifacts and challenges in accurately quantifying certain tissue characteristics. Additionally, DWI may be influenced by factors such as perfusion and inflammation, potentially affecting the reliability of the obtained images (29). Radiomic-based models, on the other hand, leverage a comprehensive set of quantitative features extracted from medical images, including texture, shape, and intensity patterns. The advantages of radiomics include its ability to capture complex spatial relationships within the tumor, potentially revealing subtle patterns that are not easily discernible through visual inspection. Radiomic features can provide a more holistic characterization of the tumor’s heterogeneity, offering a broader perspective for discrimination tasks. However, radiomic analysis requires careful standardization of imaging protocols and segmentation methods to ensure reproducibility and generalizability of results (30). Our radiomics analysis showed promising results, demonstrating a diagnostic differentiation performance that surpassed these non-radiomic approaches.

### 4.3 Determinants of performance

The identification of the most influential texture matrices, namely GLRLM and NGTDM, provides valuable insights for future radiomics model development. Both these entities provide features based on texture analysis, quantifying the spatial distribution of voxel intensities in medical images and providing information about image coarseness and homogeneity. More specifically, GLRLM focuses on the lengths of homogeneous runs of pixels with the same gray-level value (31), while, NGTDM focuses more on the local intensity differences without considering specific patterns (32). By incorporating these features into radiomics models, researchers can potentially identify specific imaging patterns associated with disease characteristics, leading to more accurate diagnoses and personalized treatment approaches.

The choice of imaging modality also plays a critical role in the performance of radiomics models. While 18F-FDG-PET/CT demonstrated the highest accuracy of 97%, its practicality and accessibility may be limited in some clinical settings (16). The 18F-FDG-PET/CT provides valuable metabolic information, making it highly effective in identifying active tumor regions. However, the need for specific on-site equipment and specialized facilities to produce the radiotracer and ensure radiation safety could restrict its accessibility in certain medical centers or regions. In contrast, MRI is more widely available and does not involve ionizing radiation, making it a safer and more convenient option for many patients.

Within the MRI techniques, the CE-T1W sequence stands out as the best-performing modality, with reported accuracies ranging from 87% to 95%. This enhanced contrast imaging modality allows for better visualization and characterization of lesions and tumor boundaries. Furthermore, the CE-T1W sequence is commonly used in clinical practice for the diagnosis and monitoring of most tumors, making it a familiar and easily interpretable imaging modality.

Cross-validation analyses were widely used in the majority (92%) of the included studies, demonstrating a rigorous approach to validating radiomics models. By dividing the dataset into multiple subsets (folds) and training the model on one subset while testing it on the remaining, cross-validation helps to simulate the model’s performance on new data. This process helps in estimating the generalization capabilities of the model and provides insights into potential overfitting issues. Its widespread use indicates a commitment to producing reliable and reproducible radiomics results, which is crucial for successful implementation in clinical settings (33). Furthermore, 33% of the studies employed also an external validation fold, pursuing further the generalization capabilities of the model.

### 4.4 Limitations

Despite the number of patients included in our study, this meta-analysis was based on retrospective cohort studies, and thus, it has limitations inherent to retrospective studies. Given the bivariate model of the meta-analysis, we did not calculate the overall ACC for the differentiation task. Moreover, our subgroup analysis was limited by the number of studies identified. It is important to acknowledge the inherent risk of selection bias, information bias, and potential confounding variables that are typical in such studies. While retrospective analyses provide valuable insights, the inability to control certain variables poses challenges in establishing causal relationships.

Nonetheless, to the best of our knowledge, this meta-analysis represents the first comprehensive synthesis of the current performance of radiomics for discriminating between CNS gliomas and PCNSLs. The findings provide cutting-edge insights to guide the development of future models.

Additionally, it is essential to recognize that remains a limitation regarding the assessment of 18F-FDG-PET/CT. We acknowledge that only one study contributed to the evaluation of this modality. Therefore, while our results suggest a high performance for 18F-FDG-PET/CT, it is crucial to interpret this with caution due to the limited data available for this modality. The systematic review also included a risk of bias assessment using the NOS. The NOS allowed for the evaluation of the quality of the included studies based on selection criteria, comparability of the study, and outcome assessment. This assessment ensured that the included studies were reliable.

## 5 Conclusions

The current SEN and SPE of radiomics to discriminate CNS gliomas from PCNSLs are high, making radiomics a helpful method to differentiate these tumor types. The best-performing texture matrices are the GLRLM, NGTDM, and the best performing features are shape-based. The 18F-FDG-PET/CT imaging modality was the best-performing, while the MRI CE-T1W was the most used and the second best-performing. There is significant heterogeneity among the current models that underscores the need for a focused developmental phase. Radiomics laboratories should be oriented towards more defined and shared algorithms that will possibly be implemented in clinical practice. Our findings suggest that integrating radiomics into diagnostic workflows may contribute to improved accuracy in distinguishing between CNS gliomas and PCNSLs.

## Data availability statement

The original contributions presented in the study are included in the article/supplementary material. Further inquiries can be directed to the corresponding author.

## Author contributions

AG: Data curation, Investigation, Writing – original draft, Writing – review & editing. NA: Data curation, Investigation, Writing – original draft. FP: Data curation, Formal analysis, Writing – original draft. PP: Supervision, Validation, Writing – review & editing. WB: Supervision, Validation, Writing – review & editing. MF: Conceptualization, Project administration, Supervision, Validation, Visualization, Writing – review & editing. LM: Conceptualization, Methodology, Project administration, Supervision, Validation, Visualization, Writing – original draft, Writing – review & editing.
